# Using a cervical ripening balloon to penetrate the placenta and quickly reduce bleeding by pressing against the placenta during pregnancy termination for patients with placenta previa in the second trimester: Two cases report

**DOI:** 10.1097/MD.0000000000022499

**Published:** 2020-09-25

**Authors:** Chang Su, Danqing Chen

**Affiliations:** Obstetrical Department, Women's Hospital,School of Medicine, Zhejiang University, Hangzhou, China.

**Keywords:** cervical ripening balloon, placenta previa, pregnancy termination, second trimester

## Abstract

**Introduction:**

The clinical treatment is complicated for patients with placenta previa who must terminate pregnancy due to fetal malformation, death, or inevitable abortion in the second trimester. It is difficult to manage excessive bleeding during pregnancy termination; and those patients face risks of removing the uterus, infection and other complications.

**Patient concerns:**

Two patients had placenta previa in the second trimester. Both cases had to terminate pregnancy. Case 1 patient had intrauterine fetal death. Case 2 patient had life-threatening vaginal bleeding. Both patients had bleeding and their cervix was not mature during vaginal delivery.

**Diagnosis:**

After hospitalization, placenta previa was confirmed by magnetic resonance imaging for case 1 patient. Placenta previa was confirmed by ultrasound examination for case 2 patient. Both patients had to terminate pregnancy.

**Interventions:**

We designed a new procedure using a cervical ripening balloon to reduce the risks during pregnancy termination for patients with placenta previa. A cervical ripening balloon was inserted through the placenta and placed between the fetus and placenta; external force was applied to keep the cervical ripening balloon pressing against the placenta that covers the cervical os. The cervical ripening balloon dilated the cervix, quickly reduced bleeding, and induced vaginal delivery during pregnancy termination for patients with placenta previa. This method was applied to 2 patients with placenta previa who must terminate pregnancy.

**Outcomes:**

Using the new method, both patients had a successful pregnancy termination and vaginal delivery with minimal bleeding. Total time from the balloon placement to the end of the delivery was about 3 hours. The procedure only used a cervical ripening balloon without uterine artery embolization needed. The fetus was delivered through the vagina; and the uterus was fully retained. There was no postpartum infection.

**Conclusion:**

This new method using a cervical ripening balloon could be a quick and effective way to reduce the risks during pregnancy termination for patients with placenta previa. It is especially helpful in emergency situations with minimal requirements of personnel and equipment. Our study showed great potential of this new utilization of a cervical ripening balloon, and is worthy of further research.

## 1 Introduction

Placenta previa is an obstetric condition when the placenta partially or completely covers the cervical os. The incidence rate of placenta previa earlier in pregnancy (approximately 24 weeks) is 4% to 5%.^[1]^ It occurs approximately in 1 of every 200 pregnancies globally. Mainland China has the highest prevalence of placenta previa in the world, at an average of 12.2 per 1000 pregnancies.^[2]^ Risk factors for placenta previa include multiple abortion history, uterine cavity operation, puerperal infection, older age, cesarean section, multiple births, poor living habits (eg. smoking, drugs), twin pregnancy, assisted reproductive technology, and abnormal uterine morphology. The exact cause of placenta previa is unclear, and may be related to placental abnormalities, endometrial lesions or injuries, and developmental delay in the fertilized egg trophoblast.

Placenta previa can cause serious bleeding and other complications that lead to morbidity and mortality during pregnancy. Occasionally, patients with placenta previa need to terminate the pregnancy due to severe fetal malformations, stillbirth, or inevitable abortion in the second trimester. It is challenging for clinicians to manage massive hemorrhage and choose the appropriate delivery method during pregnancy termination for such patients. Vascular interventional techniques have been utilized to reduce bleeding due to placental previa. However, those techniques usually associate with embolism complications such as ovarian dysfunction, pain, fever and infection. Uterine artery embolization is a complicated and expensive procedure that requires a radiology surgical team and equipment. In severe cases, hysterotomy abortion or even hysterectomy is still needed.

This study here only used a cervical ripening balloon in 2 patients with placenta previa who underwent pregnancy termination in the second trimester. This new method not only rapidly stopped bleeding but also gently increased the cervical opening. Both patients safely delivered the fetus through the vagina in a short period of time with minimal bleeding.

**Figure 1:**
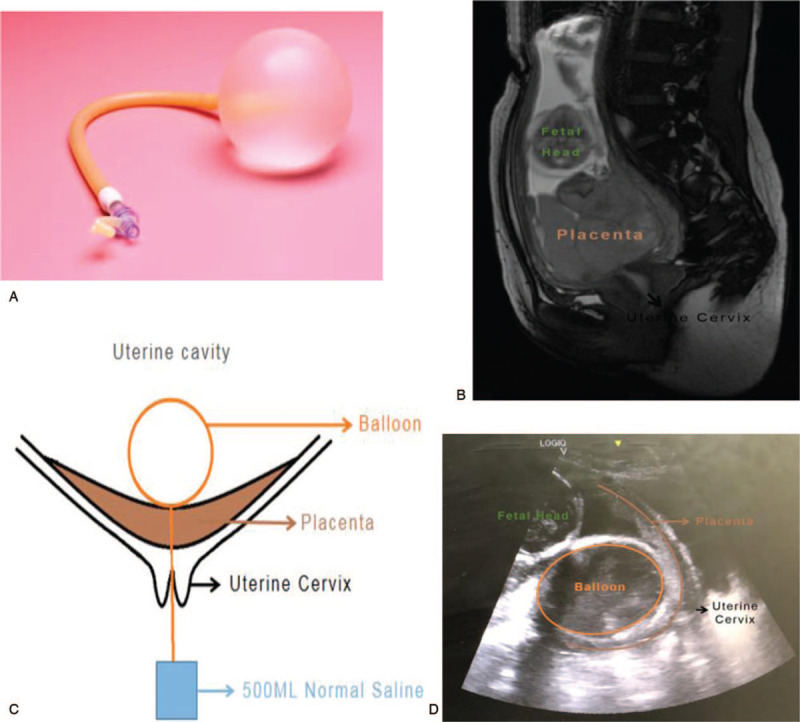
Technical details of using a cervical ripening balloon during pregnancy termination for patients with placenta previa. 1A. A cervical ripening balloon was used. 1B. The exact position of the placenta was evaluated via pre-procedural MRI. 1C. A cervical ripening balloon is inserted through the placenta using toothless oval forceps and was placed in between the fetus and placenta. After the balloon end plug is closed, external traction force (a bag of 500 mL saline) was connected to the balloon to maintain the tension for the cervical ripening balloon to press against the placenta that covers the cervical os. 1D. The positional relationship between the cervical ripening balloon and the placenta is evaluated again by ultrasound. MRI = magnetic resonance imaging.

The following are the technical details of using a cervical ripening balloon (Fig. 1A) to manage hemorrhage and promote cervical opening during pregnancy termination for patients with placenta previa. The exact position of the placenta is evaluated via pre-procedural magnetic resonance imaging (MRI) (Fig. 1B). Pregnant women are placed in the lithotomy position, the vulva is disinfected, and cervix is exposed and disinfected. Vascular forceps are inserted through the cervical os and allowed to rapidly penetrate the placenta attached to the cervix. Under ultrasound guidance, a cervical ripening balloon is carefully inserted through the placenta using toothless oval forceps and was placed in between the fetus and placenta. Certain amount of saline is injected into the cervical ripening balloon, so that the balloon diameter is similar to fetal biparietal diameter at the second trimester (150 mL saline is equal to the 6 cm of balloon diameter). After the balloon end plug is closed, external traction force (a bag of 500 mL saline) was connected to the balloon to maintain the tension for the cervical ripening balloon to press against the placenta that covers the cervical os (Fig. 1C). The positional relationship between the cervical ripening balloon and the placenta is evaluated again by ultrasound (Fig. 1D).

## 2 Case reports

This method was applied to 2 patients who had placenta previa in the second trimester. Both patients safely delivered the fetus through the vagina in a short period of time with minimal bleeding.

### 2.1 Case 1

A 26-year-old woman (gravida 1, para 0) at 24 weeks of pregnancy with placenta previa was referred to our hospital for intrauterine fetal death in March 2019. MRI on March 15, 2019 showed that the lower edge of the placenta completely covered the cervical os, with no obvious infiltration of placenta in uterine wall or cervix. The estimated thickness of the placenta at the os was 3 centimeters. After an intra amniotic injection of 100 mg ethacridine lactate solution, 50 mg mifepristone was administered orally once every 12 hours 3 times. At 18:45 on March 16, uterine contractions were normal at 3-min intervals and lasting for 25 seconds. The cervix opening was dilated to 1.5 cm. The total volume of intermittent vaginal bleeding was 200 mL (by weight) in 40 minutes. The patient's hemoglobin level was 127 g/L. Under the ultrasound guidance, a cervical ripening balloon was inserted through the placenta and placed between the fetus and the placenta using toothless oval forceps. Saline (150 mL) was injected into the cervical ripening balloon. At the time of cervix expansion, an external traction force was applied to the balloon; vaginal bleeding was immediately reduced due to the cervical ripening balloon compressing against the placenta. At 21:09, when the cervical ripening balloon was slipped out of the vagina, the cervix opening was dilated to 3 cm, with fetal presentation at S-1 cm (the lowest point of fetal skull is 1 cm above the ischial spine plane) and fetal head close to the cervix; contractions were normal. Six minutes later, the fetus was delivered via the vagina; there was total 50 mL of bleeding during the delivery. Placenta and fetal membranes were naturally delivered afterward without placental defect. The total time from the balloon placement to the end of the delivery was 2 hours and 30 minutes. The patient recovered in the hospital for 3 days with no complications. The hypersensitive C-reactive protein level was 22.2 mg/L and the hemoglobin level was 115 g/L post-delivery. The patient was discharged from the hospital on day 3 post-delivery. After 10 days, the follow-up transabdominal ultrasound showed normal endometrial thickness of 0.2 cm (single layer) and endometrial cavity width of 0.4 cm.

### 2.2 Case 2

A 30-year-old woman (gravida 2, para 0, early abortion 1) at 21 weeks of pregnancy was referred to our hospital for placenta previa with a small amount of vaginal bleeding in April 2019. Three days before hospitalization, the patient experienced dark red vaginal bleeding due to unknown etiology. Ultrasound examination indicated placenta previa. On the day of her hospital admission, ultrasound examination showed that the lower edge of the placenta covered the cervical os, and the cervical length was 2.6–2.7 cm with internal os closed. The estimated thickness of the placenta at the os was 1 cm. A 1.3 × 1.3 × 1.0 cm area with uneven flocculating echo was found between the cervical internal os and the edge of the placenta. The patient's hemoglobin level was 118 g/L. A small amount of vaginal bleeding continued after hospital admission. Two daily intravenous infusion of 25% magnesium sulfate was applied to inhibit uterine smooth muscle contraction and 1.5 g cefuroxime was administered to prevent infection. At 17:40 on April 3, 2019, the patient suffered vaginal bleeding of 380 mL. Her uterine tension was low, and the abdomen was soft. At 18:10, the cumulative vaginal bleeding volume was nearly 800 mL (by weight) with bright red color. Because the severe vaginal bleeding was possibly life threatening, the patient decided to terminate the pregnancy. Her hemoglobin level was 104 g/L and hypersensitive C- reactive protein level was 7.6 mg/L. At 19:20, the blood pressure was dropped to 87/51 mm Hg, the patient was given 3 units of red blood cell suspension and 410 mL of fresh frozen plasma. At 19:30, a cervical ripening balloon was inserted through the placenta and was placed between the fetus and placenta with ultrasound guidance. Vaginal bleeding was rapidly reduced as the external force pulled the cervical ripening balloon to press against the placenta while expanding the cervix. At 22:55, the cervical ripening balloon was slipped out of the vagina. The cervix opening was 2 cm, the fetal head was close to the cervix; contractions were normal. At 23:00, the fetus was delivered via vagina; there was 350 ml of blood loss during the delivery. The placenta and fetal membranes were naturally delivered with placental defects of 4 × 3 × 3 cubic centimeters and fetal membrane defects of 2/3. Intravenous infusion of oxytocin (20 IU) was administered to promote uterine contraction, and curettage was conducted with ultrasound guidance. There was another 380 ml of blood loss during the curettage procedure. The uterus was massaged associated with intramuscular injections of 20IU oxytocin and 250 μg carboprost tromethamine, and intravenous injections of 100 μg carbetocin. At 23:15, the bleeding stopped after using 1 piece of intrauterine iodoform gauze. On the next day, her hemoglobin level was 88 g/L. There was no bleeding after intrauterine iodoform gauze removal. Next, 1.5 g cefuroxime was intravenously infused 2 times daily for 2 days to prevent infection. The patient's temperature was normal. On post-delivery day 5, hypersensitive C- reactive protein level was 25.8 mg/L, and hemoglobin level was 86 g/L. Transabdominal ultrasound showed a 0.8 cm wide echo band in the uterine cavity with no blood flow signal. The patient was successfully discharged at post-delivery day 6.

## 3 Discussion

Placenta previa is a condition in which the placenta reaches or covers the cervical internal os.^[1]^ Many scholars believe that at the second trimester, placenta previa should be called placenta previa status^.^^[3–4]^ The placenta previa status depends on the relationship between the placenta edge and the cervical internal os. Placenta previa is normally confirmed by vaginal ultrasound, MRI, and computed tomography angiography. The sensitivity and specificity of transabdominal ultrasound is lower for the diagnosis of placental previa. Vaginal ultrasound has a high rate of accuracy for placental previa diagnosis. One study found that vaginal ultrasound helped diagnose low-lying placenta or placenta previa in nearly 1 in 10 women.^[5]^ MRI has good soft tissue contrast and clearly shows the placenta position and the depth of myometrial invasion. MRI and computed tomography can be valuable for a placenta previa diagnosis when ultrasound results are unclear.^[6]^

Placenta previa can cause serious bleeding and other complications that lead to morbidity and mortality during pregnancy. Occasionally, patients with placenta previa need to terminate the pregnancy due to severe fetal malformations, stillbirth, or inevitable abortion in the second trimester. For pregnancy termination, 1 study compared 5 ways of terminating pregnancy during the second trimester, including intra-amniotic injection of ethacridine lactate, intravenous infusion of oxytocin, cervical ripening balloon placement, and vaginal medication with prostaglandin E2 or misoprostol. The study showed that the first 3 methods were more effective than the later 2.^[7]^ Another study found that the intra-amniotic injection of ethacridine lactate combined with oral mifepristone tablets could shorten the abortion time compared with an amniotic injection of ethacridine lactate solution alone. ^[8]^ However, all these pregnancy termination methods couldn’t decrease the risk of bleeding for patients with placenta previa. Obstetric hemorrhage is the leading cause of maternal morbidity and mortality. Regardless of the method of labor induction, cervical dilatation is needed for vaginal delivery. For patients with placenta previa, the placenta is attached to the lower part of the uterus and the cervical internal os. The placenta has poor stretch ability and could detach, leading to life-threatening bleeding. Takashi Yamada et al^[9]^ reported 2 cases of hysterotomy abortion due to massive bleeding during pregnancy termination at 18 weeks. Although the pregnant woman was safe and the uterus was retained, hysterotomy abortion caused uterine scarring. The risk of placenta previa, placental implantation and massive hemorrhage is increased in the future pregnancy. ^[10]^ The interval between hysterotomy and the next pregnancy is longer. ^[11]^ Even if the hysterotomy is performed, there are still risks of uncontrolled bleeding, disseminated intravascular coagulation, shock, blood transfusion, and even hysterectomy.

Vascular interventional techniques such as uterine artery embolization, internal iliac artery balloon occlusion and abdominal aortic balloon occlusion have been utilized by obstetricians to reduce bleeding due to placenta previa. However, preventive use of any vascular intervention techniques cannot completely prevent bleeding. There are embolism complications such as ovarian dysfunction, pain, fever, and infection. In severe cases, hysterotomy abortion or even hysterectomy is still needed due to sepsis or labor induction failure.^[12]^ In the case of severe bleeding, emergency uterine artery embolization requires a radiology surgical team, additional equipment, extra time for patient transfer and operation. Bleeding may be aggravated during the process; uterine contraction medicine may lead to vasoconstriction and then increase the operational difficulties. This is a significantly challenging procedure with potential risks.^[13]^ Studies have shown that preventive uterine artery embolization does not significantly improve the outcomes of pregnancy termination in patients with complete placenta previa, but increases the risk of complications and leads to a longer hospital stay.^[14]^ Therefore, the application of vascular interventional techniques in obstetrics is a complicated and expensive procedure that also needs additional personnel and equipment. The necessity and safety of vascular interventional techniques need to be further explored.

Tang et al^[15]^ reported 3 cases of uterine artery embolization combined with double balloon catheter for patients with placenta previa who had an abortion due to fetal malformations during the second trimester. Patients undergoing induced labor suffered hemorrhage after an intra-amniotic injection of ethacridine lactate solution and oral mifepristone tablets. Uterine artery embolization was applied first, and subsequently a double balloon catheter was placed in the lower part of the uterine cavity and outside the cervical external os, the balloon was left there for 12 hours. After the cervix was opened at 2 cm, the balloon was taken out. Although the vaginal delivery was successful, the operation procedure was complicated with long induction time and increased risk of infection.

This study here only used a single cervical ripening balloon without uterine artery embolization needed. The cervical ripening balloon was inserted through the placenta and placed between the fetus and the placenta. This method not only rapidly stopped bleeding by pressing against the placenta that covers the cervical os, but also gently increased the cervical opening and induced uterine contraction. Total time from the balloon placement to the end of the delivery was about 3 hours for both patients. Both patients safely delivered the fetus through the vagina in a short period of time with minimal bleeding. For the first patient, the hemorrhage volume before placement of the cervical ripening balloon was 200 mL. The total time from the balloon placement to the end of the delivery was 2 hours and 30 minutes, with 50 mL of bleeding. For the second patient, the hemorrhage volume before placement of the cervical ripening balloon was 800 mL. The whole procedure lasted for 3 hours and 30 minutes, with 740 mL of bleeding. This new method in this study had a shorter operation time compared to other methods in previous studies. The shorter procedure time could reduce the risk of continued bleeding and infection. Even the second patient had hemorrhage and placental membrane residues after the delivery; the methods of curettage, uterine gauze packing, or uterine balloon packing could be applied to stop bleeding and preserve the integrity of the uterus.

Some complications may occur during pregnancy termination for patients with placenta previa, including uncontrolled bleeding, unsuccessful conservative treatment, and the risk of amniotic fluid embolism; hysterotomy abortion or even hysterectomy may be needed in certain situations. A study in Canada showed that the risk of amniotic fluid embolism doubled due to induction of labor.^[16]^ However, another study in the US showed that amniotic fluid embolism was not associated with labor induction.^[17]^ Another complication is intrauterine or systemic infection due to bleeding and intrauterine operations. Hysterotomy increases the risks of wound infection, endometritis and other complications. A study showed that preventive use of antibiotics does reduce the incidence of infection by 60% to 70%.^[18]^ The short operation and delivery time could possibly reduce the risk of infection. Indeed, in this study, there was no amniotic fluid embolism and infection in either patient.

This study showed that a cervical ripening balloon could be used as a simple, rapid and effective way to terminate pregnancy and manage bleeding in patients with placenta previa. The requirements for personnel and equipment are minimal for this method. It is a practical way to reduce bleeding during pregnancy termination for patients with placenta previa. The operation could be considered under emergency situations even in rural areas to minimize the risks of major hemorrhagic shock or even death during the hospital transferring. This new method can quickly stop bleeding, decrease the operation time, retain uterus integrity, and reduce the occurrence rate of hysterotomy, scarring or uterus loss. Whether this operation will increase the risk of amniotic fluid embolism remains to be further explored. Currently, there are no similar clinical operations using a cervical ripening balloon to penetrate the placenta and manage the bleeding in patients with placenta previa. Our study showed great potential for this new application of a cervical ripening balloon, and is worthy of further research.

## Acknowledgments

The authors are grateful to the patient who signed informed consent for publication.

## Author contributions

**Conceptualization:** Chang Su, Danqing Chen.

**Data curation:** Chang Su.

**Funding acquisition:** Danqing Chen.

**Investigation:** Chang Su, Danqing Chen.

**Methodology:** Chang Su, Danqing Chen.

**Project administration:** Chang Su, Danqing Chen.

**Resources:** Danqing Chen.

**Supervision:** Danqing Chen.

**Visualization:** Chang Su.

**Writing – original draft:** Chang Su.

**Writing – review and editing:** Danqing Chen.
